# Live-cell analysis of kinetochore–microtubule interaction in budding yeast

**DOI:** 10.1016/j.ymeth.2010.01.017

**Published:** 2010-06

**Authors:** Kozo Tanaka, Etsushi Kitamura, Tomoyuki U. Tanaka

**Affiliations:** a Wellcome Trust Centre for Gene Regulation and Expression, College of Life Sciences, University of Dundee, Dundee, UK; b Institute of Development, Aging and Cancer, Tohoku University, Sendai, Miyagi, Japan

**Keywords:** *Saccharomyces cerevisiae*, Chromosome, Kinetochore, Centromere, Spindle, Microtubule, Mitosis, Live cell imaging, Fluorescence microscopy

## Abstract

Kinetochore capture and transport by spindle microtubules plays a crucial role in high-fidelity chromosome segregation, although its detailed mechanism has remained elusive. It has been difficult to observe individual kinetochore–microtubule interactions because multiple kinetochores are captured by microtubules during a short period within a small space. We have developed a method to visualize individual kinetochore–microtubule interactions in *Saccharomyces cerevisiae*, by isolating one of the kinetochores from others through regulation of the activity of a centromere. We detail this technique, which we call ‘centromere reactivation system’, for dissection of the process of kinetochore capture and transport on mitotic spindle. Kinetochores are initially captured by the side of microtubules extending from a spindle pole, and subsequently transported poleward along them, which is an evolutionarily conserved process from yeast to vertebrate cells. Our system, in combination with amenable yeast genetics, has proved useful to elucidate the molecular mechanisms of kinetochore–microtubule interactions. We discuss practical considerations for applying our system to live cell imaging using fluorescence microscopy.

## 1 Introduction

Proper sister chromatid segregation to opposite poles of the cell during mitosis is crucial for the maintenance of genetic integrity in eukaryotic cells. For high-fidelity chromosome segregation, sister kinetochores must be properly captured by spindle microtubules [1], and then pulled towards opposite spindle poles after the loss of cohesion of sister chromatids at the metaphase–anaphase transition. In animal cells, kinetochores are captured by microtubules in prometaphase after nuclear envelope breakdown [2], [3], [4]. Because kinetochore capture and transport could be discerned only in a few cell types, mechanisms of these processes have remained elusive.

The budding yeast, *Saccharomyces cerevisiae*, is an excellent model organism to study the mechanism for chromosome segregation because of its amenable genetics. In addition, the development of systems to detect chromosome loci by marking them with binding sites for sequence-specific DNA binding proteins [5], [6] expanded our knowledge of the mechanism of chromosome segregation by allowing direct visualization of chromosome motion in living cells. In these systems, *lac* or *tet* operators are integrated tandemly at chromosome sites of interest, which are bound by the Lac repressor (LacI) or tetracycline repressor (TetR) fused to fluorescent proteins, respectively [5], [6]. When centromere movements were visualized, sister centromeres were found to split on the spindle when they bi-orient, while sister chromatids are still being held together by cohesion along their arms [7], [8], [9], [10]. However, it is poorly understood how kinetochores interact with microtubules before they bi-orient on the spindle.

Budding yeast undergoes a closed mitosis; the nuclear envelope remains intact throughout the cell cycle. Microtubule organizing centers, called spindle pole bodies (SPBs), are embedded in the nuclear envelope [11], [12], and kinetochores are tethered to SPBs by microtubules during most of the cell cycle, including G1 and M phases [12], [13], [14], [15]. Nonetheless, several lines of data have suggested that centromeres are released from, and recaptured by microtubules during a brief period in S phase, due to kinetochore disassembly and reassembly upon centromere DNA replication [15], [16]. However, in normal S phase, it is difficult to analyze individual kinetochore–microtubule interactions in detail, because all kinetochores interact with microtubules in the vicinity of a spindle pole where microtubules frequently overlap with each other.

To analyze the initial kinetochore–microtubule interaction in budding yeast, we have developed two assay systems, both of which are based on live cell microscopy imaging. The first assay involves delaying the assembly of a kinetochore on a particular centromere [17]. Budding yeast kinetochores assemble on short (∼130 bp) centromere sequences [18], and attach to a single microtubule [19], [20]. It was reported that centromere function can be inactivated by activating transcription from an adjacently-inserted promoter, which inhibits kinetochore assembly [21], [22]. We inserted the *GAL1-10* promoter in the vicinity of *CEN3* [22], to conditionally inactivate and activate the centromere by turning on and off transcription in the presence of galactose and glucose, respectively. This procedure prevents the centromere from associating with the mitotic spindle. While cells are arrested in metaphase, we reactivate the centromere, which leads to kinetochore assembly and interaction with microtubules extending from a spindle pole. In this system, which we have called ‘centromere reactivation system’, we found that kinetochores were captured by the lateral surface of a single microtubule extending from a spindle pole, and were subsequently transported poleward along the microtubule [17], [23]. This process of initial kinetochore capture and subsequent transport along microtubule is evolutionarily conserved from yeast to vertebrate cells [2], [3], [4], [17].

In the second assay, we observe kinetochore–microtubule interactions without artificial regulation of kinetochore assembly or without a cell cycle arrest [24]. Upon centromere DNA replication, kinetochores are disassembled, which causes centromeres to detach from microtubules and move away from a spindle pole. Subsequently (1–2 min later) kinetochores are reassembled and captured by microtubules (kinetochore–microtubule interaction in normal S phase).

The first assay allows observation of the kinetochore–microtubule interactions with higher spatial resolution because the centromere moves away from a spindle pole and other centromeres for longer distances. On the other hand, the second assay enables analyses in a more physiologic condition. We can gain both advantages by comparing outcomes from both assays to analyze the mechanisms underlying the initial kinetochore–microtubule interaction.

In this article, we mainly focus on the centromere reactivation system as a method to dissect the mechanisms of kinetochore–microtubule interaction in budding yeast. We first introduce the technical details of the method and then show the applications of this system to elucidate various aspects of kinetochore–microtubule interactions. We also describe the method to observe kinetochore–microtubule interaction in normal S phase. We will not explain how to analyze chromosome dynamics after sister kinetochore bi-orientation is established or after cells enter anaphase, which has been described elsewhere [7], [8], [9], [10]. Finally, we summarize the current view on kinetochore–microtubule interactions during the cell cycle in budding yeast.

## 2 ‘Centromere reactivation system’ in budding yeast

We replaced *CEN3* on chromosome III with *CEN3* under control of the *GAL1-10* promoter [22], to conditionally inactivate and activate the centromere by turning on and off transcription in the presence of galactose and glucose, respectively. For live cell imaging by fluorescence microscopy, we labeled the *CEN3*–adjacent sequence and α-tubulin (*TUB1*) with fluorescent proteins to visualize *CEN3* and microtubules, respectively [5]. We inactivated the *CEN3* and simultaneously arrested cells in metaphase by depleting Cdc20, which is required for sister chromatid separation and for anaphase onset [25]. In this situation, *CEN3* localizes away from the spindle and is well separated from all other centromeres attached to the spindle. Then, we reactivated *CEN3* by turning off the adjacent *GAL1-10* promoter while cells were still in metaphase, and followed the behavior of *CEN3*.

### 2.1 Yeast strain

The basic yeast strain we used for centromere reactivation system (T3531 [17]) has the following genotype with the W303 strain background: *MATa*, *cdc20∷Pmet3-CDC20∷TRP1*, *cen3∷Pgal-CEN3-tetOs∷URA3*, *leu2∷TetR-GFP∷LEU2*, *trp1∷YFP-TUB1∷TRP1*. Each construct in T3531 was obtained as follows.

#### 2.1.1 Replacement of the authentic CEN3 with Pgal-CEN3-tetOs

**Fig. 1:**
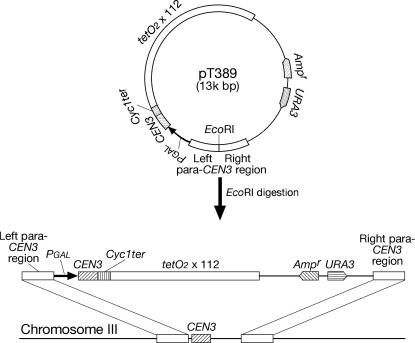
Replacement of *CEN3* with the *Pgal-CEN3* construct for the centromere reactivation system. The linearized plasmid pT389 (see Section 2.1.1) was integrated into the *CEN3* locus through recombination at para-*CEN3* regions. *CEN3*, para-*CEN3* regions, *GAL1-10* promoter, *CYC1* transcription terminator, an array of *tetOs*, ampicillin-resistant gene, and *URA3* gene are indicated.

First, *Pgal-CEN3* [22], *CYC1* transcription terminator (350 bp amplified by PCR) and 112 tandem copies of *tetOs* [5] (spanning 5.6 kb) were cloned into YIplac211 (National Centre for Biotechnology Information X75462) in the above order; second, the left and right *CEN3*-flanking regions (about 1 kb; not containing *CEN3* itself) were amplified by PCR and cloned next to the *GAL1-10* promoter in the above plasmid (at the opposite side from *CEN3*) with the opposite orientation (joining their 3′ ends together) (pT389); third, pT389 was cut between two para-*CEN3* DNA fragments and used for transformation of yeast cells (Fig. 1).

#### 2.1.2 Other constructs

*TetR-GFP* [5] and *Pmet3-CDC20* [26] were previously reported. *YFP-TUB1* plasmid (pDH20) were obtained from Yeast Resource Center (Seattle, USA) and used for yeast cell transformation (integrated at *TRP1* locus).

### 2.2 Yeast cell culture

**Fig. 2:**
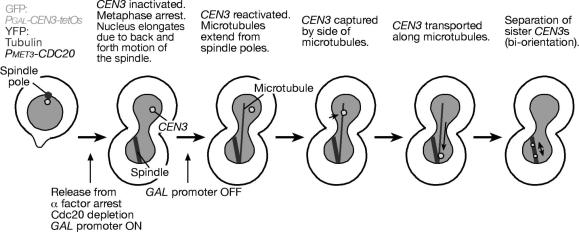
Diagrams of the experimental system for observing kinetochore capture by microtubules (centromere reactivation system) and the process of kinetochore capture and transport. See Sections 2, 3 for details.

(1) Culture *Pmet3-CDC20 Pgal-CEN3-tetOs TetR-GFP YFP-TUB1* cells (T3531) in synthetic complete (SC)-met medium + 2% raffinose at 25 °C overnight. SC-met medium is made by dissolving 6.7 g of yeast nitrogen base w/o amino acids (Becton Dickinson 291940) and 770 mg of CSM-Met (Foremedium DCS0111) in deionised water, then add 1 ml of 1 M NaOH and 10 ml of 1% adenine to 950 ml in total, followed by autoclaving to sterilize. We usually prepare 30 ml of culture in a 100 ml flask, and culture cells in a shaking water bath with rotation at 160 rpm.
(2) Adjust OD600 of cell culture to 0.2–0.3. Treat cells with *α*-factor (13 aa peptide: WHWLQLKPGQPMY) to arrest cells in G1 phase. Add 5 mg/ml *α*-factor stock solution in methanol to a final concentration of 0.5 μg/ml at *t* = 0, 1 and 2 h. Check for proper arrest under a microscope 20–30 min after the last *α*-factor addition.
(3) Pour cell culture onto a membrane filter (Whatman ME28 10 400 812; diameter 47 mm, pore size 1.2 μm) set in a vacuum filtration device to remove *α*-factor. Wash cells three times with YPA medium or distilled water. YPA medium is made by dissolving 20 g of peptone (Becton Dickinson 211677) and 10 g of yeast extract (Becton Dickinson 212750) in deionised water, then add 10 ml of 1% adenine (Sigma A8751) to 950 ml in total, followed by autoclaving. Take the membrane filter with cells on it from the filtration device and place into YPA medium + 2% raffinose + 2% galactose + 2 mM methionine (Sigma M9625) in a flask. We usually use 40 ml medium in a 100 ml flask. Resuspend cells by shaking.
(4) Release cells from G1 arrest in a shaking water bath at 25 °C. Cdc20 is depleted in the presence of methionine while *CEN3* is inactivated in the presence of galactose. Depending on the purpose, cells can be directly arrested at metaphase from asynchronous culture. As it is not necessary to wash out *α*-factor in this case, dilute culture in SC-met medium + 2% raffinose into more than two volumes of YPA medium + 2% raffinose + galactose (final 2%) + methionine (final 2 mM). Cells arrest at metaphase with large buds in 2 h. When cells are arrested at metaphase for longer (>3 h), nuclei are often elongated between buds and mother cell bodies (Fig. 2). This elongation is probably due to earlier back-and-forth motions of the spindle between the two cell bodies, and is not due to cells leaking into anaphase, because the spindle length stays short. Typically, inactivated *CEN3* remains in the mother while the short spindle is found in the bud in the elongated nucleus (Fig. 2). If *CEN3* and the spindle are located on the opposite sides of the elongated nucleus, *CEN3* capture by long microtubules can be observed in detail.

### 2.3 Sample preparation for microscopy

For microscopy, cells are mounted either on microscope slides or on glass-bottom dishes depending on the purpose. Cells on microscope slides become somewhat flattened under the cover slip, enabling most of the cells to be kept within a single microscope field and on the same focal plane. On the other hand, cells sometimes change their location during imaging when the mounting medium dries up. In addition, this method may negatively affect cell viability, especially when images are collected over an extended-time period. Mounting cells on glass-bottom dishes has the opposite attributes; cells within a single microscope field are not usually on the same focal plane, but their viability is maintained for longer. When observing temperature-sensitive cells at their restrictive temperature, the objective lens and glass-bottom dishes need to be heated.

#### 2.3.1 Live cell imaging on microscope slides

**Fig. 3:**
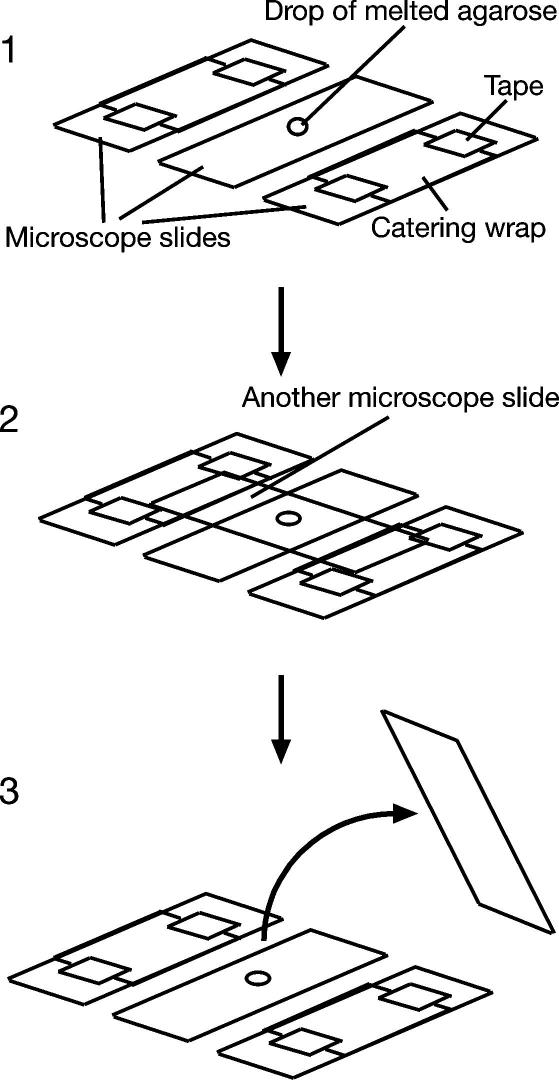
Making agarose pads on microscope slides. See Section 2.3.1 for details.
(2) Take 0.5–1.0 ml of cell culture. Spin down by centrifugation at 5000 rpm for 30 s at room temperature. Wash twice with 1 ml of SC medium. Resuspend in a small volume of SC medium + 2% glucose + 2 mM methionine. Now *CEN3* is reactivated by the addition of glucose and begins to be captured by microtubules. Therefore, the following procedures should be done as quickly as possible (within 5 min).
(3) Mount 2–3 μl of cell suspension on the agarose pad on a microscope slide. Cover cells with a cover slip (VWR 631-0125; 22 × 22 mm, thickness no. 1.5). Gently tap edges of the cover slip to spread cell suspension. Cells tend to be trapped on the agarose pad while medium spreads to the edge of the cover slip.
(4) Set the microscope slide on the stage of a microscope and find a field suitable for imaging. In our experience, cells at the edge of the agarose pad are well concentrated and do not move even when medium dries up in other areas.

(1) Prepare microscope slides with agarose pads. Boil 5% agarose (Invitrogen Ultra Pure for Molecular Biology 15510-027) in water in a microwave and then allow to cool to 68 °C in a heat block. Heat also SC medium (×1.5 concentration) + 3% glucose to 68 °C in a heat block. SC medium is made by dissolving 6.7 g of yeast nitrogen base w/o amino acids (Becton Dickinson 291940) and 790 mg of CSM (Foremedium DCS0011) in deionised water, then add 1 ml of 1 M NaOH and 10 ml of 1% adenine to 950 ml in total, followed by autoclaving. Mix one volume of 5% agarose to two volumes of SC medium + 2% glucose. To make the pad, prepare two slides covered by a piece of catering wrap (taped to the slide; Fig. 3). Place a clean slide (VWR Superfrost 631-0103) between these two slides. Drop 3 μl of medium/agarose onto the center of the slide (Fig. 3, step 1), and quickly place another slide perpendicularly on top of the first slide so that its ends are supported by the catering wrap (Fig. 3, step 2). The thickness of the catering wrap prevents the pad being too thin between the two slides. Remove the top slide soon after agarose becomes hard (∼10 min), leaving the pad on the bottom slide (Fig. 3, step 3). These microscope slides with agarose pads can be used for a couple of days.

#### 2.3.2 Live cell imaging on glass-bottom dishes

(1) Coat a glass-bottom dish with Concanavalin A (Sigma C7275). Apply 100 μl of 0.2% Concanavalin A to the microwell of a MatTek dish (MatTek Corporation P35G-1.5-10-C; dish diameter 35 mm, glass thickness no. 1.5, microwell diameter 10 mm). Place in the dark for 10 min. Remove liquid and dry the dish in the dark.
(2) Take 0.5–1.0 ml of cell culture. Spin down by centrifugation at 5000 rpm for 30 s at room temperature. Wash with 1 ml of SC medium + 2% raffinose + 2% galactose. Resuspend in 0.2–0.5 ml of SC medium + 2% raffinose + 2% galactose.
(3) Mount 100 μl of cell suspension in the microwell of a Concanavalin A-coated MatTek dish on the stage of a microscope. Wait 7 min then remove medium. To wash out galactose and remove floating cells, wash the dish by 1 ml of SC medium twice (carefully add and then immediately remove the medium by pipetting).
(4) Mount 1 ml of SC medium + 2% glucose + 2 mM methionine to reactivate *CEN3*.

#### 2.3.3 Live cell imaging of temperature-sensitive mutant strains

##### 2.3.3.1 On microscope slides

(1) Heat objective lens to 35 °C with an objective heater system (Bioptechs) following the manufacturer’s instructions. Heat microscope slides with agarose pads to 35 °C on a heating block. Also heat SC medium for washing and SC medium + 2% glucose + 2 mM methionine to 35 °C on a heating block.
(2) Transfer the cell culture in YPA + 2% raffinose + 2% galactose + 2 mM methionine from a shaking water bath at 25 °C to another water shaker bath at 35 °C prior to imaging.
(3) After incubation at 35 °C for a period required to inactivate a mutant (typically 30 min), take samples, then wash and mount cells as in Section 2.3.1, except for using pre-heated medium and microscope slides.

##### 2.3.3.2 On glass-bottom dishes

(1) Use Delta T culture dishes (Bioptechs 04200415C; 0.17 mm thick clear) for which a heating device is available, instead of MatTek dishes. To coat the glass surface of the dishes with Concanavalin A, mount 150 μl of 0.2% Concanavalin A (sufficient to cover the middle of the glass surface). Incubate for 10 min in the dark, remove liquid and dry the dishes.
(2) Heat the objective lens with the objective heater system (Bioptechs Delta T open culture dish system) and heat a Concanavalin A-coated Delta T culture dish with the dish heater system to 35 °C. Also heat SC medium + 2% raffinose + 2% galactose and SC medium without carbon source for washing and SC medium + 2% glucose + 2 mM methionine to 35 °C in a heating block.
(3) Transfer the cell culture in YPA + 2% raffinose + 2% galactose + 2 mM methionine from a shaking water bath at 25 °C to one at 35 °C prior to imaging.
(4) After incubation in a shaking water bath at 35 °C for a required period to inactivate a mutant, take samples, then wash the cells as in Section 2.3.2, but use pre-heated medium.
(5) Mount 150 μl of cell suspension in the microwell of a Concanavalin A-coated Delta T culture dish on the stage of a microscope. Wait 7 min and remove medium. To wash out galactose and remove floating cells, wash the dish twice using 1 ml of pre-heated SC medium, carefully adding the medium and removing immediately by pipetting.
(6) Mount 2 ml of pre-heated SC medium + 2% glucose + 2 mM methionine to reactivate *CEN3*.

### 2.4 Fluorescence microscopy

As the signal intensity from fluorescent proteins expressed in yeast is very weak mainly due to small number of the molecules in this small organism, a wide-field microscope which can collect more signal is advantageous over a confocal microscope. Three-dimensional live cell imaging followed by deconvolution makes the signal clearer and brighter.

#### 2.4.1 Image collection

Images are taken using an inverted microscope (Olympus IX-71 in Deltavision microscope (Applied Precision)) with a 100 × 1.4 numerical aperture optical lens, a cooled CCD camera (Photometrics CoolSNAP HQ) and softWoRx software (Applied Precision). In typical experiments, time-lapse images are collected every 10–15 s for 20–40 min with 5–9 (0.5–0.7 μm apart) *z*-sections at 23 °C (ambient temperature) or 35 °C (for temperature-sensitive cells). To distinguish GFP and YFP signals in time-lapse fluorescence microscopy, the JP3 filter set (Chroma) is used. In typical experiments, exposure time to excitation light is 0.1 s for both GFP/JP3 and YFP/JP3 channels with appropriate neutral density filters, but this depends on the signal intensity of target samples.

#### 2.4.2 Image analysis

Acquired images are deconvoluted and projected with maximum intensity signals to two-dimensional images with the softWoRx software.

## 3 Studying kinetochore–microtubule interactions using the centromere reactivation system

**Fig. 4:**
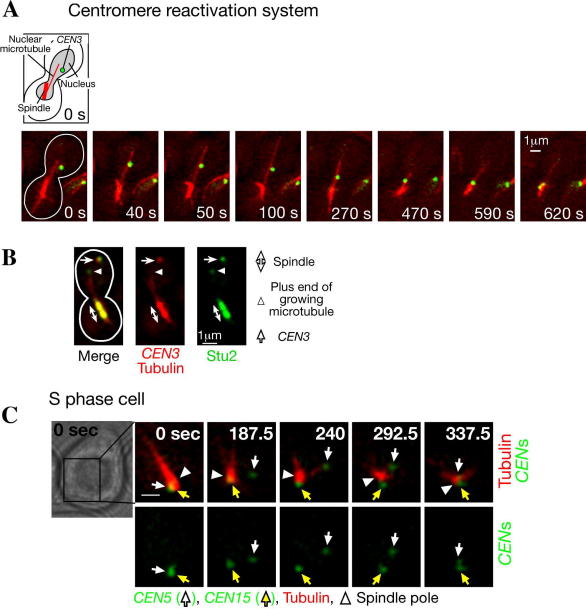
Examples of live cell imaging. (A) Visualizing kinetochore capture by microtubules in the centromere reactivation system. *Pmet3-CDC20 Pgal-CEN3-tetOs TetR-GFP YFP-TUB1* cells (T3531) [17] were treated and observed as described in Section 2. Zero time is set arbitrarily for the first panel, in which the cell shape is outlined. Scale bar, 1 μm. (B) Localization of Stu2 on kinetochores and microtubules, visualized using the centromere reactivation system. *STU2-3GFP Pmet3-CDC20 Pgal-CEN3-tetOs TetR-3CFP CFP-TUB1* cells (T3680) [17] were treated as in (A), except that CFP (*CEN3* and tubulin) and GFP (Stu2) signals were collected separately. CFP and GFP signals are shown in red and green, respectively. Scale bar, 1 μm. (C) Visualizing kinetochore detachment from microtubules, recapture, and poleward transport in a normal cell cycle [24]. *CEN5-tetOs TetR-3CFP CEN15-lacOs GFP-LacI YFP-TUB1* cells (T4243) [24] were treated with *α*-factor and subsequently released to fresh medium. After 30 min, CFP/GFP and YFP images were collected every 7.5 s for 8 min. *CEN5* and *CEN15* are shown in green, and microtubules are shown in red. White arrows, yellow arrows and white arrowheads indicate *CEN5*, *CEN15* and a spindle pole, respectively. 0 s in the montage; start of image acquisition. Scale bar; 1 μm.

Using the centromere reactivation system, we found that the kinetochore is captured by the side of microtubules, often a single microtubule, and transported in a manner to that shown in animal cells [17]. Cells with unattached *CEN3*s positioned at distance from spindles in elongated nuclei are suitable for analysis of kinetochore capture by long microtubules (Fig. 2, Fig. 4A, 0 s). *CEN3*s are captured by microtubules within 20 min after addition of glucose in most of the cells. *CEN3*s are usually captured by the side of the microtubules (Fig. 4A, 40 s), and subsequently transported along the microtubules (Fig. 4A, 100–590 s). Shortly after *CEN3*s reach a spindle pole, the majority of *CEN3*-GFP signals split indicating that sister *CEN3*s bi-orient on the metaphase spindle (Fig. 4A, 620 s).

Figure C is adapted from a figure in Ref. [24], with permission.

### 3.1 Dissecting molecular mechanisms of kinetochore capture

In the centromere reactivation system, we can divide the kinetochore capture process into several steps [17] (Fig. 2). First, microtubules extend from spindle poles. Next, kinetochores are captured by the side of a microtubule. Then, kinetochores are transported along the microtubule towards a spindle pole. Finally, after becoming bi-oriented the sister kinetochores separate, and are pulled towards opposite spindle poles. With our centromere reactivation system, we combined mutants that are known to cause chromosome missegregation. By observing these strains for defects in kinetochore capture, we could identify molecules involved in each step of the process, described in Ref. [17].

### 3.2 Analyzing dynamics of nuclear microtubules

One advantage of our centromere reactivation system is that we can clearly see individual microtubules in elongated nuclei. Under normal circumstances microtubules appear only as part of a spindle bundle. This affords the opportunity to analyze the dynamic behaviors of nuclear microtubules, as has been done for cytoplasmic microtubules [27], [28]. To follow microtubule dynamics in detail, we need to shorten the time interval for image capture in live cells (3–5 s). This can be achieved by reducing the number of *z*-sections (three sections per time point) and capturing both *CEN3*-GFP and YFP-tubulin signal simultaneously using the YFP channel of the JP4 filter set (Chroma). It is possible to distinguish nuclear microtubules from cytoplasmic microtubules by labeling the nuclear rim with Nic96 (a component of the nuclear pore complex) fused with YFP [29]. Nuclear microtubules can also be identified by the presence of Dam1 [23] or absence of Kip2 [30] fused with three tandem copies of GFP along the microtubules (see below).

We could identify molecules involved in the dynamics of nuclear microtubules, and found that capture of kinetochores by microtubules facilitates microtubule rescues (conversion from shrinkage to growth) [17]. We also found two mechanisms of microtubule-dependent poleward kinetochore transport [23]. First, kinetochores slide along the microtubule lateral surface (lateral sliding). Second, kinetochores are tethered at the microtubule distal ends and pulled poleward as microtubules shrink (end-on pulling).

### 3.3 Determine the localization of molecules on kinetochores or nuclear microtubules

#### 3.3.1 Investigating kinetochore localization of molecules of interest

In normal conditions, yeast kinetochores cluster together on the spindle, and it is difficult to discriminate one kinetochore from the others. Although we can assume that certain molecules localize on kinetochores by observing their colocalization with the cluster of centromere signals, it is difficult to analyze localization of certain molecules on individual kinetochores. As the centromere reactivation system enables us to isolate one of the sister kinetochore pairs from others, we can reliably judge the localization of molecules on individual kinetochores. For this purpose we use the centromere reactivation system strains containing *TetR-3CFP* (Tet repressors fused with three tandem copies of cyan fluorescent protein (CFP)) [31] and *CFP-TUB1* instead of *TetR-GFP* and *YFP-TUB1*, respectively [17], [23]. Molecules of interest are tagged with three tandem repeats of GFP at their genomic loci [32], [33]. If the tagged molecules localize to kinetochores, GFP signal is seen on *CEN3* after centromere reactivation in the YFP channel of the JP4 filter set, while *CEN3* and tubulin are seen in the CFP/JP4 channel. As an example, the kinetochore localization of Stu2, a microtubule plus end-tracking protein (+TIPs) [34], is shown in Fig. 4B.

#### 3.3.2 Investigating localization on nuclear microtubules

The centromere reactivation system is also useful to investigate the localization of molecules of interest on nuclear microtubules using strains as described above. By analyzing the dynamics of a molecule along nuclear microtubules during their polymerization and depolymerization, we can characterize the behavior of the molecules on nuclear microtubules [17], [23]. In Fig. 4B, Stu2 is seen at the plus end of growing microtubule, consistent with its property as a +TIP member [34].

## 4 Visualizing kinetochore–microtubule interactions in normal cell cycle

### 4.1 Visualizing kinetochore–microtubule interactions in S phase

Recent indirect evidence has suggested that kinetochores might be transiently disassembled during S phase, causing centromeres to detach from microtubules [16], [17], [35]. Such centromere motion has been overlooked in the past, probably because it happens for such a short time period. By setting a very short time interval for image acquisition we have visualized this process [24].

#### 4.1.1 Yeast strain

To study centromere behavior with time-lapse microscopy, we marked *CEN5* and *CEN15* by the adjacent insertion of a *tet* or *lac* operator array [7], [10], respectively. These arrays were bound by Tet repressors fused with three tandem copies of CFP (TetR-3CFP) [31], and by LacI with a single copy of GFP (GFP-LacI) [6]; thus *CEN5* and *CEN15* were visualized as small CFP and GFP dots, respectively. Microtubules were also visualized by the expression of α-tubulin (*TUB1*) fused with YFP (YFP-TUB1).

#### 4.1.2 Yeast cell culture

(1) Culture *CEN5-tetOs TetR-3CFP CEN15-lacOs GFP-LacI YFP-TUB1* cells (T4243) in YPA + 2% glucose at 25 °C overnight.
(2) Synchronize cells in G1 phase with *α*-factor treatment as shown in Section 2.2.
(3) Release cells into fresh YPA + 2% glucose medium (see Section 2.2) and culture at 25 °C in YPA + 2% glucose for 15 min followed by sample preparation for microscopy (see Section 2.3.2); note that all media in Section 2.3.2 is replaced with SC medium + 2% glucose.

#### 4.1.3 Live cell imaging and analysis

To follow the trajectory of *CEN*s in detail, CFP/GFP and YFP images are collected every 7.5 s for 8 min with 5 (0.7 μm apart) *z*-sections (see Section 2.4.1); note that using the JP3 filter set, CFP/GFP and YFP are visualized separately, and the two *CEN*s can be distinguished because *CEN15*-GFP shows higher intensity than *CEN5*-CFP. To avoid false judgements, detachment of GFP- and CFP-labeled *CEN*s from YFP-labeled microtubules is scored only when *CEN* signals do not overlap with microtubule signals for two or more consecutive time points. Detachment is also scored when *CEN*-spindle pole distance is 700 nm or larger. Moreover, detachment is not scored if the *CEN* moved only along the *z* axis, as resolution along the *z* axis is not as good as on the *x*–*y* plane.

Both *CEN5* and *CEN15* stayed in the vicinity of a spindle pole (<0.5 μm from the center of the pole) during G1 phase (Fig. 4C, 0 s). However, just before bud emergence, which corresponds to early S phase, both *CEN*s detached from microtubules and moved away from a spindle pole (Fig. 4C, 187.5 s shows *CEN5* detachment); note that SPBs have not yet separated and cells have a single spindle pole in S phase [36]. While *CEN*s are detached (for 1–2 min), their distance from the spindle is on average 1.0 μm. Centromere detachment from microtubules was found to be dependent on its DNA replication, which results in disassembly of kinetochores. Kinetochores are reassembled soon afterward (Ref. [24], see Fig. 6), and recaptured by microtubules (Fig. 4C, 240 s shows *CEN5* capture). Kinetochores are subsequently transported poleward by microtubules to the vicinity of a spindle pole, where they stay thereafter (Fig. 4C, 240–337.5 s show *CEN5* transport). Kinetochores are transported in two ways, lateral sliding and end-on pulling, which are distinguished by observing localization of the Dam1 complex component Ask1 on *CENs*, in a similar manner as seen in the centromere reactivation system [23].

### 4.2 Overview of kinetochore–microtubule interactions in budding yeast during the cell cycle

**Fig. 5:**
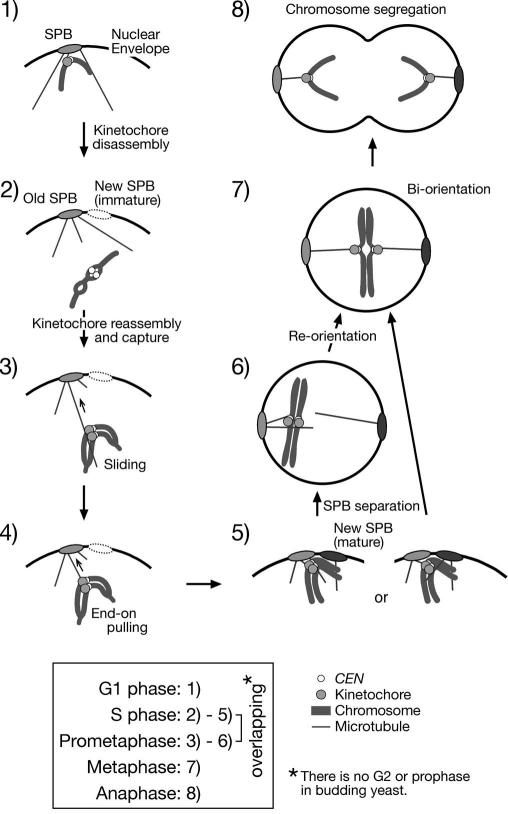
Overview of kinetochore–microtubule interactions during the cell cycle in budding yeast. Only a part of the nuclear envelope is shown in (1)–(5), while the whole is depicted in (6)–(8). See Section 4.2 for details.

The current view on kinetochore–microtubule interactions in budding yeast during the cell cycle is as follows [24], [37]:

(1) During G1 phase, SPBs are embedded in the nuclear envelope and centromeres are tethered at SPBs via microtubules [13], [14], [15] (Fig. 5, step 1).

The figure is adapted from a figure in Ref. [24].
(2) Centromeres become transiently (between 1 and 2 min) detached from microtubules in S phase, leading to centromere displacement away from a spindle pole (Fig. 5, step 2). Centromere detachment and displacement are dependent on centromere DNA replication in early S phase. Our data suggest that upon centromere DNA replication, kinetochores are disassembled, causing centromere detachment from microtubules.
(3) Centromeres are subsequently reassembled after DNA replication, leading to centromere recapture by microtubules (Fig. 5, step 3). The old SPB, which has been inherited from the previous cell cycle, usually organizes microtubules for centromere recapture (Fig. 5, step 3), while the new SPB, which is formed *de novo* in the vicinity of the old SPB is immature. Centromeres are initially captured by the lateral surface of microtubules [17] (Fig. 5, step 3).
(4) Subsequently, centromeres are transported poleward in two distinct ways: lateral sliding and end-on pulling [23] (Fig. 5, steps 3 and 4). Sliding is often converted to end-on pulling but the opposite conversion is rare.
(5) By microtubule-dependent poleward transport, kinetochores reach the vicinity of a spindle pole, where the microtubule density becomes higher, thus allowing both sister kinetochores to interact with other microtubules more efficiently (Fig. 5, step 5). Meanwhile the new SPB becomes mature enough to organize microtubules, and the Ipl1 and Mps1 kinases facilitate re-orientation of kinetochore–microtubule attachment [15], [38]. Re-orientation might involve transient detachment of centromeres from microtubules. The Ipl1- and Mps1-dependent re-orientation of kinetochore–microtubule attachment is regulated in a tension-dependent manner [39]. Thus, after SPBs separate and form a bipolar spindle at the end of S phase [36], this re-orientation promotes sister kinetochore bi-orientation that generates tension at kinetochores (Fig. 5; steps 6 and 7).
(6) When all sister kinetochore pairs establish bi-orientation, Scc1 in the cohesin complex, which holds sister chromatids together, is cleaved, which leads cells into anaphase (Fig. 5; step 8). The spindle elongates and sister chromatids segregate towards opposite SPBs, probably transported by end-on pulling [23].

In the open mitosis of metazoan cells, there is a large temporal gap (G2 phase) between DNA replication and the initial kinetochore–microtubule interaction, because spindle poles must wait for nuclear envelope breakdown in order to organize microtubules for kinetochore capture [40]. By contrast, in the closed mitosis of budding yeast, spindle poles are connected to kinetochores via microtubules throughout most of the cell cycle [12], [13], [14], becoming detached only for a short period in S phase. Considering that centromeres are recaptured by microtubules already during S phase, we propose that in budding yeast, (1) there is no G2 phase (and no prophase) and (2) S phase and M phase (prometaphase) significantly overlap (Fig. 5).

In spite of such difference, the mechanisms underlying kinetochore–microtubule interactions are remarkably similar in early mitosis (prometaphase and metaphase) [37], [41], [42] between budding yeast and metazoan cells. In both, centromeres are initially captured by the lateral surface of microtubules and transported poleward along microtubules by minus-end directed motors; subsequently the Aurora B/Ipl1 kinase facilitates sister kinetochore bi-orientation, which is monitored by a conserved mechanism of the spindle checkpoint; both bi-orientation and spindle-checkpoint mechanisms respond to tension applied on kinetochores, for which the conserved cohesin complex is required.

## 5 Concluding remarks

Kinetochore capture and bi-orientation are fundamental cellular events during mitosis. We therefore expect that many aspects of the underlying mechanisms will be conserved from yeast to vertebrates, even if modifications were added during the evolution process. This is proving to be the case as is recently reported (reviewed in Ref. [37]). Recent advances in fluorescence microscopy have made yeast cells, which are much smaller than animal cells, available for detailed analysis of kinetochore–microtubule interactions. Further investigation in budding yeast is expected to uncover more regulatory mechanisms of kinetochore capture and transport, which are relevant to mitosis in all eukaryotic cells.
